# CRL4^WDR1^ Controls Polo-like Kinase Protein Abundance to Promote Bilobe Duplication, Basal Body Segregation and Flagellum Attachment in *Trypanosoma brucei*

**DOI:** 10.1371/journal.ppat.1006146

**Published:** 2017-01-04

**Authors:** Huiqing Hu, Qing Zhou, Xianxian Han, Ziyin Li

**Affiliations:** Department of Microbiology and Molecular Genetics, McGovern Medical School, University of Texas Health Science Center at Houston, Houston, Texas, United States of America; University of York, UNITED KINGDOM

## Abstract

The Polo-like kinase homolog in *Trypanosoma brucei*, TbPLK, plays essential roles in basal body segregation, flagellum attachment and cytokinesis. The level of TbPLK protein is tightly controlled, but the underlying mechanism remains elusive. Here, we report a Cullin-RING ubiquitin ligase composed of Cullin4, the DNA damage-binding protein 1 homolog TbDDB1 and a WD40-repeat protein WDR1 that controls TbPLK abundance in the basal body and the bilobe. WDR1, through its C-terminal domain, interacts with the PEST motif in TbPLK and, through its N-terminal WD40 motif, binds to TbDDB1. Depletion of WDR1 inhibits bilobe duplication and basal body segregation, disrupts the assembly of the new flagellum attachment zone filament and detaches the new flagellum. Consistent with its role in TbPLK degradation, depletion of WDR1 causes excessive accumulation of TbPLK in the basal body and the bilobe, leading to continuous phosphorylation of TbCentrin2 in the bilobe at late cell cycle stages. Together, these results identify a novel WD40-repeat protein as a TbPLK receptor in the Cullin4-DDB1 ubiquitin ligase complex for degrading TbPLK in the basal body and the bilobe after the G1/S cell cycle transition, thereby promoting bilobe duplication, basal body separation and flagellum-cell body adhesion.

## Author Summary

African trypanosomes are the causative agents of sleeping sickness in humans and nagana in cattle in sub-Saharan Africa. These unicellular parasites possess a flagellum, which is a long, lash-like appendage that is protruded from the cell body and serves for locomotion of the parasites. The flagellum is attached to the cell body via a specialized structure termed flagellum attachment zone (FAZ) filament. Assembly of the FAZ filament is necessary to maintain flagellum-cell body attachment, which is required for maintaining normal cell shape and for ensuring faithful cell division. A key regulator of flagellum-cell body attachment is the Polo-like kinase (PLK), an evolutionarily conserved protein kinase that regulates many downstream effectors. The level of PLK appears to be tightly controlled, but the underlying mechanism is still elusive. Here we report a novel protein that controls PLK abundance by targeting PLK for degradation. Depletion of this novel protein causes excessive accumulation of PLK and disrupts FAZ filament assembly, leading to flagellum detachment and defective cell division. These results discover the molecular mechanism underlying the control of PLK protein abundance in trypanosomes.

## Introduction

Polo-like kinases (PLKs) are evolutionarily conserved serine/threonine protein kinases and play multiple essential roles in mitotic entry, centrosome maturation, spindle assembly, chromosome segregation, mitotic exit and cytokinesis in a wide variety of eukaryotes [1,2]. PLKs are characterized by an N-terminal kinase domain and a C-terminal Polo-box domain (PBD) consisting of two Polo-box motifs, which mediates PLK localization through binding to PLK-phosphorylated docking proteins at various subcellular locations [3,4,5]. The abundance of PLK protein is under tight regulation through the ubiquitin-proteasome pathway. Degradation of human PLK1 and the budding yeast PLK homolog Cdc5 is mediated by the anaphase-promoting complex/cyclosome (APC/C) in conjugation with the cofactor Cdh1, and requires a destruction box (D-box) [6,7]. Degradation of human PLK4, a regulator of centriole duplication, is mediated by the Cullin-RING ligase (CRL) SCF^β-TrCP/Slimb^ [8,9], and requires a PEST motif that is trans-phosphorylated by PLK4 itself [10,11]. The PEST motif is a stretch of peptide rich in proline (P), glutamic acid (E), serine (S) and threonine (T), and is well known as a signal sequence for protein degradation [12]. PLK4 thus controls its own stability through trans-autophosphorylation of the PEST motif to create a phospho-degron that is recognized by the F-box protein β-TrCP/Slimb for ubiquitination and degradation [13,14].

The parasitic protozoan *Trypanosoma brucei* contains a well-conserved PLK homolog, TbPLK, which, structurally, resembles human PLK1 but, functionally, is distinct from PLK1. TbPLK contains an N-terminal kinase domain and a C-terminal PBD consisting of two Polo-box motifs, and displays a dynamic localization during the cell cycle [15,16,17]. During G1 phase, TbPLK localizes to the basal body and a bilobed structure, which is adjacent to the proximal end of the newly assembled flagellum attachment zone (FAZ) filament. When the new FAZ filament is assembled near the bilobe during S phase, TbPLK disappears from the basal body and the bilobe, but instead localizes to the distal tip of the extending FAZ filament. Following cell cycle progression, TbPLK remains at the new FAZ tip before it disappears at late anaphase [18]. TbPLK plays diverse roles in bilobe duplication, basal body rotation and segregation, FAZ assembly and cytokinesis initiation in the procyclic form [15,16,19], and appears to be required for cleavage furrow ingression in the bloodstream form [19]. By identifying and characterizing several TbPLK substrates, such as TbCentrin2 [16], SPBB1 [20], and CIF1 [18], the mechanistic roles of TbPLK have started to be uncovered. TbCentrin2 is phosphorylated by TbPLK in the bilobe during G1 and S phases, but appears to be dephosphorylated thereafter. Dephosphorylation of TbCentrin2 is necessary for bilobe duplication, FAZ assembly and flagellum attachment [21]. SPBB1 localizes to the basal body, and is required for basal body segregation, FAZ assembly and flagellum attachment by functioning as a downstream effector of TbPLK [20]. CIF1 localizes to the distal tip of the new FAZ filament, which is TbPLK dependent, and regulates cytokinesis initiation by targeting the Aurora B kinase TbAUK1, an essential cytokinesis regulator in *T*. *brucei* [22,23], to the new FAZ tip during late anaphase for TbAUK1 to drive cytokinesis initiation [18].

Although we have learned a great deal about the physiological functions of TbPLK, our knowledge about the spatiotemporal control of TbPLK activity and abundance during the cell cycle is still limited. The finding that overexpression of TbPLK caused a severe cell growth defect [19] suggests that TbPLK protein level is under tight control. TbPLK changes its location from the basal body and the bilobe to the new FAZ tip during the cell cycle transition from G1 to S phase [16], and disappears from the new FAZ tip at late anaphase [18]. It is unclear whether the basal body- and bilobe-localized TbPLK is degraded or transfers to the new FAZ tip when the new FAZ is assembled during S phase. However, the disappearance of TbPLK from the new FAZ tip at late anaphase suggests that FAZ tip-localized TbPLK is probably degraded.

In this report, we identified a Cullin4-based ubiquitin ligase complex CRL4^WDR1^, which mediates TbPLK degradation in the basal body and the bilobe. WDR1 is a WD40-repeat protein and acts as a TbPLK receptor in the CRL4 ubiquitin ligase complex. Depletion of WDR1 impaired TbPLK ubiquitination and degradation, leading to excessive accumulation of TbPLK in the basal body and the bilobe and continuous phosphorylation of the bilobe protein TbCentrin2 after S phase. WDR1 deficiency disrupted bilobe duplication, basal body segregation, FAZ assembly and flagellum attachment, reminiscent of ectopic TbPLK overexpression. These findings revealed the mechanism underlying the stringent control of TbPLK protein abundance in the bilobe and the basal body to ensure bilobe duplication, basal body segregation and flagellum-cell body adhesion.

## Results

### TbPLK is a short-lived protein and its degradation requires a PEST motif and three D-boxes

**Fig 1:**
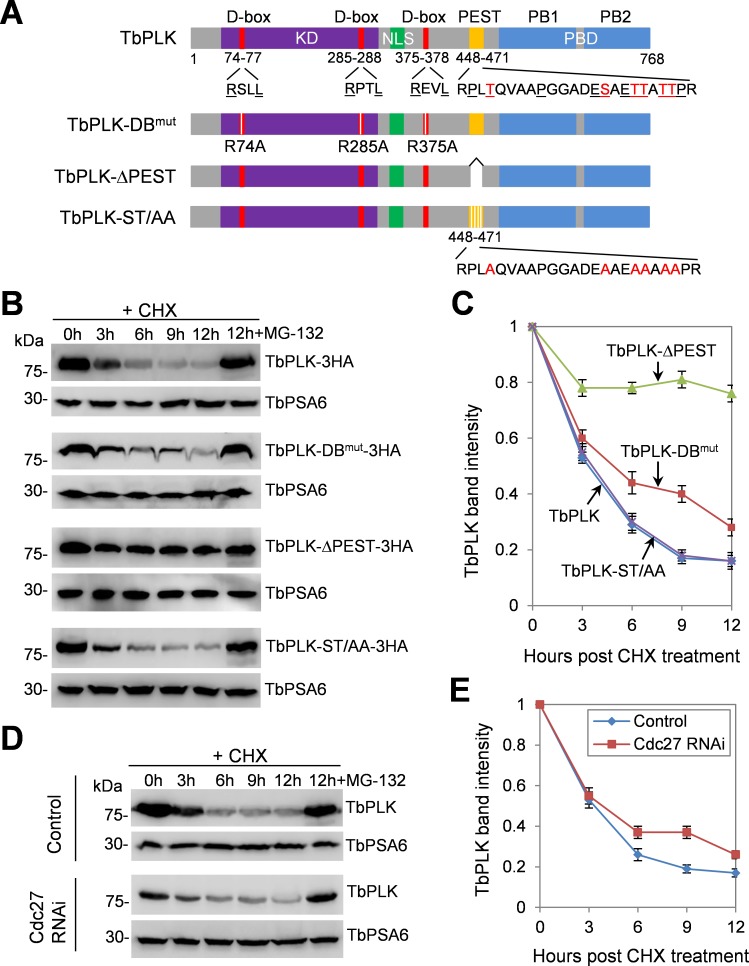
Degradation of TbPLK is mediated by destruction box and PEST motif. (**A**). Schematic drawing of the domains in TbPLK and the various mutants of TbPLK expressed for protein stability assay. DB, destruction box or D-box; KD, kinase domain; NLS, nuclear localization signal sequence; PEST, proline (P), glutamic acid (E), serine (S) and threonine (T)-enriched sequence; PBD, Polo-box domain; PB1 and PB2, Polo-boxes 1 and 2. The signature arginine (R) and leucine (L) residues in the three D-boxes, and the proline (P), glutamate (E), serine (S) and threonine (T) residues in the PEST motif are underlined. The phosphorylated serine and threonine residues in the PEST motif are highlighted in red. (**B**). Degradation of ectopically overexpressed TbPLK and its D-box mutant and PEST-deletion mutant. Cells overexpressing 3HA-tagged TbPLK or each of the TbPLK mutants were treated with cycloheximide, and time-course samples (equal numbers of cells) were collected for Western blotting with anti-HA antibody. In a separate cell sample, MG-132 was added together with cycloheximide and incubated for 12 h (12h+MG-132). TbPSA6, the *T*. *brucei* proteasome subunit alpha-6, served as the loading control. (**C**). Quantification of TbPLK band intensity from panel B. TbPLK band intensity was measured with ImageJ, and normalized with the band intensity of TbPSA6. Error bars represent S.D. calculated from three independent experiments. (**D**). Degradation of TbPLK in control and Cdc27 RNAi cells, which was induced for 72 h. Cells were treated with cycloheximide for up to 12 h, and time-course samples were collected for Western blotting with anti-TbPLK antibody. The level of TbPSA6 served as the loading control. (**E**). Quantification of TbPLK band intensity from panel D. TbPLK band intensity was measured with ImageJ, and normalized with the band intensity of TbPSA6. Error bars indicate S.D. calculated from three independent experiments.

TbPLK contains two potential degradation motifs, putative D-boxes at amino acids 74~77, 285~288 and 375~378, and a putative PEST motif at amino acids 448~471 (Fig 1A). Within the PEST motif, a number of serine and threonine sites are phosphorylated by unknown protein kinase(s) *in vivo* [24] (Fig 1A). The D-box is best known to be recognized by the APC/C ubiquitin ligase, whereas the PEST motif is often recognized by the CRL-type ubiquitin ligase. To investigate whether TbPLK is a short-lived protein and to identify the motif(s) responsible for TbPLK degradation, we mutated the essential arginine residues R74, R285 and R375 in the three putative D-boxes to alanine to make a D-box mutant (TbPLK-DB^mut^), and deleted the putative PEST motif to make a PEST deletion mutant (TbPLK-ΔPEST) (Fig 1A). We then tagged the two TbPLK mutants and wild-type TbPLK with a triple HA epitope and overexpressed them in *T*. *brucei*. These overexpressed wild-type and mutant TbPLK proteins all localized to the basal body, the bilobe and the new FAZ tip, although TbPLK-DB^mut^ was additionally detected in the cytosol (S1A Fig). Subsequently, we carried out pulse-chase experiments and Western blotting with anti-HA antibody to monitor the degradation of 3HA-tagged TbPLK and TbPLK mutants. We found that TbPLK was gradually degraded in cells treated with cycloheximide, with its half-life estimated to be ~3.5 h (Fig 1B and 1C). In the presence of the proteasome inhibitor MG-132, TbPLK was stabilized (Fig 1B), indicating that TbPLK is degraded by the proteasome. TbPLK-DB^mut^ was still degraded, albeit at a slightly slower rate than TbPLK, with an estimated half-life of ~4.5 h (Fig 1B and 1C). The level of TbPLK-ΔPEST was reduced to ~80% after cycloheximide treatment for 3 h, but was not further reduced thereafter (Fig 1B and 1C). To test whether phosphorylation of the PEST motif contributes to TbPLK degradation, we mutated the phospho-serine and phospho-threonine sites in the PEST motif to alanine to make a phospho-deficient mutant (TbPLK-ST/AA) (Fig 1A). TbPLK-ST/AA was degraded at a rate similar to TbPLK (Fig 1B and 1C). These results suggest that degradation of TbPLK depends on the D-boxes and the PEST motif, but does not require phosphorylation of the PEST motif. Further, a D-box and PEST double mutant, TbPLK-DB^mut^-ΔPEST, was overexpressed, which localized to the basal body, the bilobe and the new FAZ tip, and additionally was detected in the cytosol (S1A Fig). Pulse-chase experiments showed that the level of TbPLK-DB^mut^-ΔPEST was reduced to ~90% of the control level after cycloheximide treatment (S1B and S1C Fig), which appeared to be degraded at a slightly slower rate than TbPLK-ΔPEST (Fig 1B and 1C). This result further confirmed that TbPLK degradation depends on both the D-boxes and the PEST motif.

To confirm that native TbPLK is a short-lived protein, pulse-chase experiment was similarly carried out, and Western blotting with anti-TbPLK antibody was performed to monitor TbPLK protein level. The results showed that TbPLK was gradually degraded (Fig 1D and 1E), similar to the overexpressed TbPLK (Fig 1B and 1C). Given that mutation of the D-boxes slightly slowed down TbPLK degradation, we examined whether APC/C is involved in TbPLK degradation. TbPLK level was examined in the procyclic cells depleted of Cdc27, an essential APC/C subunit in *T*. *brucei* [25], and the results showed that the degradation rate of TbPLK in Cdc27 RNAi cells was slightly slower than that in the non-induced control cells (Fig 1D and 1E) and was similar to that of TbPLK-DB^mut^ (Fig 1B and 1C). These results suggest that APC/C is involved in TbPLK degradation, but it is not the sole and the major ubiquitin ligase responsible for TbPLK degradation. Given that deletion of the PEST motif significantly slowed down TbPLK degradation (Fig 1B and 1C), it suggests that a specific CRL-type ubiquitin ligase is the major ubiquitin ligase for TbPLK degradation.

### Identification of the CRL4^WDR1^ complex that interacts with TbPLK

**Fig 2:**
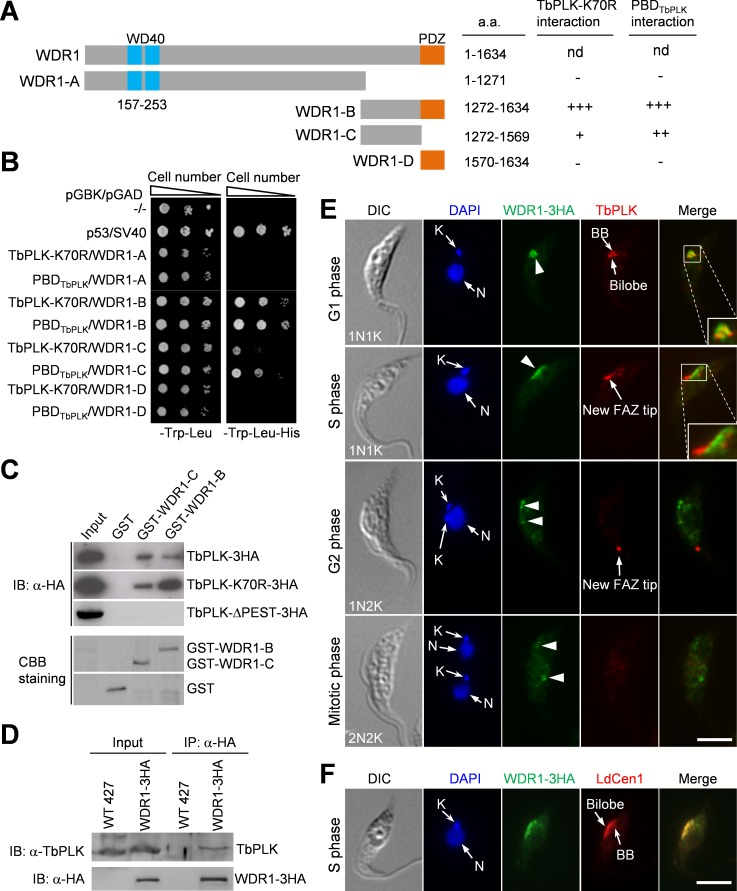
WDR1 interacts with TbPLK through its C-terminal domain and partially overlaps with TbPLK in the basal body and the bilobe at G1 phase. (**A**). Schematic drawing of the domains in WDR1 and the truncation mutants of WDR1, and summary of yeast two-hybrid results. nd, not done because we failed to clone the full-length *WDR1* gene into the yeast expression vectors despite multiple attempts. (**B**). Directional yeast two-hybrid assay to detect the interaction between TbPLK-K70R, PBD_TbPLK_ and WDR1 truncation mutants. (**C**). WDR1, through its C-terminal domain, interacts with the PEST motif of TbPLK *in vitro*. The truncation fragments of WDR1 were expressed as GST-fusion proteins in *E*. *coli*, purified and used to pull down TbPLK-3HA, TbPLK-K70R-3HA, and TbPLK-ΔPEST-3HA from *T*. *brucei* cell lysate. CBB, coomassie brilliant blue. (**D**). WDR1 interacts with TbPLK *in vivo* in *T*. *brucei*, as demonstrated by co-immunoprecipitation. Endogenously 3HA-tagged WDR1 was immunoprecipitated with anti-HA antibody conjugated to protein G sepharose beads, and immunoprecipitated proteins were immunoblotted with anti-TbPLK antibody and anti-HA antibody to detect TbPLK and WDR1-3HA, respectively. (**E**). Subcellular localization of WDR1 and TbPLK during the cell cycle. Cells expressing endogenously 3HA-tagged WDR1 were co-immunostained with FITC-conjugated anti-HA mAb and anti-TbPLK pAb, and counterstained with DAPI for nuclear (N) and kinetoplast (K) DNA. Scale bar: 5 μm. (**F**). Localization of WDR1 to the basal body and the bilobe region during the S phase of the cell cycle. Cells were co-immunostained with FITC-conjugated anti-HA mAb to label WDR1-3HA and anti-LdCen1 pAb to label the basal body and the bilobe. Among the 115 S-phase cells examined, all of them showed WDR1-3HA localization to the basal body and the bilobe region. Scale bar: 5 μm.

We recently carried out yeast two-hybrid library screening with a kinase-dead mutant TbPLK, TbPLK-K70R, and the PBD (a.a. 434~768 including the PEST motif) of TbPLK as baits, and identified several TbPLK-interacting proteins and potential substrates [20]. We further characterized one of these TbPLK partners, which is encoded by Tb927.10.15080 and contains two WD40 repeats at the N-terminus and a PDZ (Post synaptic density protein, *D**rosophila* disc large tumor suppressor, and Zonula occludens-1 protein) domain at the C-terminus (Fig 2A). We named it WDR1 for WD40 Repeat 1. In the original yeast two-hybrid library screen [20], WDR1 was identified by the PBD but not the TbPLK-K70R. However, in the directional yeast two-hybrid assay, the C-terminal domain of WDR1 interacts with both the PBD and TbPLK-K70R (Fig 2A and 2B). It appears that as the bait, TbPLK-K70R had lower efficiency than PBD in yeast two-hybrid library screening [20]. Nevertheless, WDR1 interacts with TbPLK via its C-terminal domain, but the PDZ domain is not required for binding to TbPLK, as demonstrated by directional yeast two-hybrid and GST pull-down assays (Fig 2A–2C). Deletion of the PEST motif in TbPLK disrupted its interaction with WDR1 (Fig 2C), suggesting that the PEST motif is required for WDR1 binding. Immunoprecipitation of WDR1 was capable of pulling down TbPLK from *T*. *brucei* cell lysate (Fig 2D), indicating that the two proteins interact *in vivo* in *T*. *brucei*.

To determine the subcellular localization of WDR1, it was endogenously tagged with a triple HA epitope. Immunofluorescence microscopy showed that in G1 and S phases WDR1-3HA was enriched in the basal body and the bilobe region, but during G2 and mitotic phases the level of WDR1-3HA appeared to be much reduced, albeit the protein was still detectable in the basal body and the bilobe region (Fig 2E). WDR1-3HA partially overlapped with TbPLK in G1 cells, but not in S-phase cells in which TbPLK was detected mainly at the new FAZ tip (Fig 2E). Co-immunostaining with anti-LdCen1, which detects TbCentrin4 in the basal body and the bilobe [26], confirmed that WDR1 localized to the basal body and the bilobe region during S phase of the cell cycle (Fig 2F).

**Fig 3:**
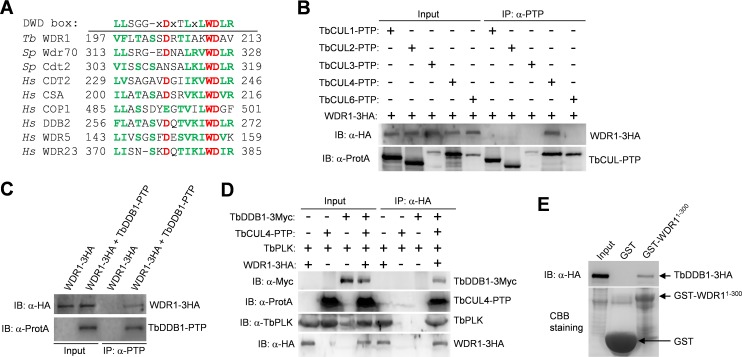
WDR1 forms a complex with TbCUL4 and TbDDB1. (**A**). Alignment of the putative DWD box in WDR1 with the DWD box of fission yeast and human WD40-repeat proteins that have been confirmed to bind to DDB1. The three highly conserved residues are highlighted in red, and other conserved residues are in green. The consensus sequence of the DWD box is shown at the top of the aligned sequences. Tb, *T*. *brucei*; Sp, *Schizosaccharomyces pombe*; Hs, *Homo sapiens*. (**B**). WDR1 interacts with TbCUL4 but not other Cullin proteins, in *T*. *brucei*, as demonstrated by co-immunoprecipitation. WDR1-3HA and each of the five PTP-tagged Cullin proteins were co-expressed from their respective endogenous locus in *T*. *brucei*. Immunoprecipitation was performed by incubating the cell lysate with IgG beads, and immunoprecipitated proteins were then immunoblotted with anti-HA antibody and anti-Protein A (α-ProtA) antibody, respectively. (**C**). WDR1 interacts with TbDDB1 *in vivo* in *T*. *brucei*, as demonstrated by co-immunoprecipitation. WDR1-3HA and TbDDB1-PTP were co-expressed in *T*. *brucei*, and immunoprecipitation and Western blotting were performed as described in panel B. (**D**). WDR1, TbCUL4, TbDDB1 and TbPLK form a complex in *T*. *brucei*, as demonstrated by co-immunoprecipitation. WDR1-3HA, TbCUL4-PTP and TbDDB1-3Myc were co-expressed from their respective endogenous locus in *T*. *brucei*. Immunoprecipitation of WDR1-3HA was carried out by incubating the cell lysate with EZview Red anti-HA affinity gel, and immunoprecipitated proteins were immunoblotted with anti-HA antibody, anti-Myc antibody, anti-TbPLK antibody and anti-Protein A (α-ProtA) antibody to detect WDR1-3HA, TbDDB1-3Myc, TbPLK and TbCUL4-PTP, respectively. (**E**). The N-terminal domain of WDR1 mediates the interaction with TbDDB1, as demonstrated by *in vitro* GST pull-down. The N-terminal fragment (1–300 aa) of WDR1, which contains the WD40 motif, was expressed as a GST-fusion protein in *E*. *coli*, purified and used to pull down TbDDB1-3HA from *T*. *brucei* cell lysate. Purified GST-WDR1^1-300^ and GST were stained by coomassie brilliant blue (CBB).

Given that WDR1 depletion significantly stabilizes TbPLK (see below), we speculated that WDR1 might function as a TbPLK receptor in a Cullin-containing ubiquitin ligase complex. In yeast and animals, Cullin-RING ubiquitin ligases include the SCF ubiquitin ligase, which is composed of Cullin1, Skp1 and an F-box protein, and the CRL4^WDR^ ubiquitin ligase, which is composed of Cullin4, DDB1 and a DDB1-binding WD40-repeat protein [27,28,29,30]. These DDB1-binding WD40-repeat proteins all contain a conserved motif termed DWD (DBB1-binding WD40 protein) box [29]. The DWD box is a stretch of 16 amino acids, which is defined by the presence of three highly conserved residues, Asp7, Trp13 and Asp14, five hydrophobic residues, and a conserved arginine residue at position 16 [29]. WDR1 also possesses a putative DWD box, which contains the three highly conserved residues (Asp204, Trp210 and Asp211) and five hydrophobic residues, but lacks the arginine residue at the last position of the DWD box (Fig 3A). It should be noted that human COP1 and WDR5 (Fig 3A) and several other WDR proteins [30] also lack this arginine residue, suggesting that this residue is not absolutely required for the function of the WDR proteins.

The trypanosome genome encodes a Cullin4 homolog (Tb927.3.1290), which we named TbCUL4 (S2 Fig), and a homolog of DDB1 (Tb927.6.5110), which we named TbDDB1 (S3 Fig). We tested whether WDR1 interacts with TbCUL4 and TbDDB1 *in vivo* in trypanosomes by co-immunoprecipitation, which showed that immunoprecipitation of TbCUL4 or TbDDB1 was able to co-precipitate WDR1 from *T*. *brucei* cell lysate (Fig 3B and 3C). Immunoprecipitation of other Cullin proteins, TbCUL1 (Tb927.11.11430), TbCUL2 (Tb927.10.7490), TbCUL3 (Tb927.8.5210) and TbCUL6 (Tb927.4.4760), did not co-precipitate WDR1 from *T*. *brucei* cell lysate (Fig 3B), suggesting that WDR1 forms a complex with TbCUL4 but not other Cullins. These results are in agreement with the findings in yeast and humans that the Cullin4-DDB1 complex contains a WD40-repeat protein [27,28,29,30]. Finally, to test whether TbCUL4, TbDDB1, WDR1 and TbPLK form a complex *in vivo* in trypanosomes, TbCUL4 was tagged with a PTP epitope, TbDDB1 was tagged with a triple Myc epitope, and WDR1 was tagged with a triple HA epitope in a single cell line. Immunoprecipitation of WDR1 was able to co-precipitate TbCUL4, TbDDB1 and TbPLK, which was detected by anti-Protein A antibody, anti-Myc antibody, and anti-TbPLK antibody, respectively (Fig 3D), confirming that the four proteins indeed form a complex in trypanosomes.

To test whether the N-terminal WD40 motif in WDR1 is sufficient for binding to TbDDB1, we purified a recombinant N-terminal truncation fragment of WDR1 containing the two WD40 repeats, and carried out GST pull-down experiment, which showed that this N-terminal truncation fragment of WDR1 was able to pull down TbDDB1 (Fig 3E). Given that WDR1, through its C-terminal domain, interacts with TbPLK (Fig 2A–2D), these results suggest that WDR1, through its N-terminal WD40 motif, binds to TbDDB1 of the TbCUL4-TbDDB1 complex and may act as a TbPLK receptor in the TbCUL4-TbDDB1 complex to mediate TbPLK degradation. We named this complex CRL4^WDR1^ for Cullin-RING Ligase 4 with WDR1 as a substrate receptor according to suggested nomenclature [31].

### Depletion of WDR1 inhibits basal body segregation and causes flagellum detachment

**Fig 4:**
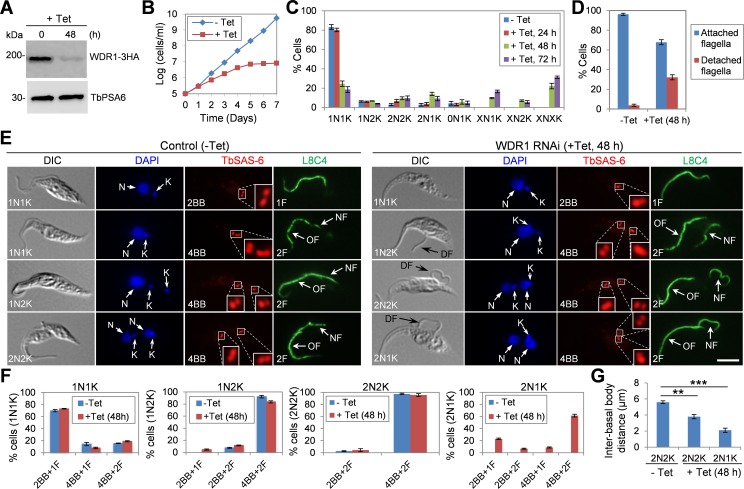
WDR1 depletion inhibited basal body segregation and caused flagellum detachment. (**A**). Western blotting to monitor the level of WDR1-3HA, which was endogenously tagged in the WDR1 RNAi cell line, before and after tetracycline induction of WDR1 RNAi. The level of TbPSA6 was included as the loading control. (**B**). RNAi of WDR1 caused a severe growth defect. Cells harboring the WDR1 RNAi construct were incubated with (+Tet) or without (-Tet) tetracycline and counted every day for 7 days. Shown is the result from one of the four clonal RNAi cell lines that target two different sequences of the *WDR1* gene. (**C**). Quantification of cells with different numbers of nucleus (N) and kinetoplast (K) before and after WDR1 RNAi. 200 cells were counted for each time point. Error bars represent S.D. (**D**). Percentage of cells with attached or detached flagellum before (-Tet) and after (+Tet) WDR1 RNAi induction for 48 h. 200 cells were counted from each time point, and error bars represent S.D. (**E**). Effect of WDR1 depletion on basal body segregation and flagellum attachment. Cells were immunostained with anti-TbSAS-6 pAb and L8C4 (anti-PFR2 mAb) to label the basal body (BB; including mature BB and pro-BB) and the flagellum, respectively. Black arrows in the DIC channel indicate the detached flagellum (DF). Scare bar: 5 μm. (**F**). Quantification of basal body (BB) and flagellum in non-induced control (-Tet) and WDR1 RNAi cells (+Tet, 48 h). 200 cells of each cell type were counted, and error bars indicate S.D. (**G**). Inter-basal body distances of 2N2K and 2N1K cells before and after WDR1 RNAi. WDR1 RNAi was induced for 48 h, and non-induced control and WDR1 RNAi cells were immunostained with YL 1/2 and anti-TbSAS-6 antibodies. Distance between the two pairs of mature basal body/pro-basal body was measured with the ImageJ software. 200 cells of each cell type from control and WDR1 RNAi were counted, and error bars indicate S.D. from three independent experiments. Statistical analysis was performed using t-test in Excel. **, *p*<0.01; ***, *p*<0.001.

To investigate the function of WDR1, we carried out RNAi in the procyclic form of *T*. *brucei*. Two different DNA fragments from the coding region of *WDR1* gene were each cloned into the RNAi vector pZJM, and two clonal cell lines from each RNAi cell line were analyzed. All four clonal RNAi cell lines showed almost identical phenotypes, and only the results from one clonal cell line were presented in this report. To monitor the efficiency of RNAi, WDR1 was endogenously tagged with a triple HA epitope in the RNAi cell line and detected by Western blotting with anti-HA antibody. Induction of RNAi with tetracycline resulted in a significant decrease of WDR1 protein level after 48 h (Fig 4A). This reduction in WDR1 level caused a severe growth defect (Fig 4B), indicating that WDR1 is essential for cell proliferation in the procyclic form. To characterize the potential defects in cell cycle progression, we quantified the number of cells with different numbers of nucleus and kinetoplast. Upon WDR1 RNAi induction, there was an increase of cells with two nuclei and one kinetoplast (2N1K) and the emergence of cells with multiple (>2) nuclei and one kinetoplast (XN1K), multiple (>2) nuclei and two kinetoplasts (XN2K), and multiple (>2) nuclei and multiple (>2) kinetoplasts (XNXK) (Fig 4C). These results suggest defects in kinetoplast segregation and cytokinesis. Moreover, microscopic analyses showed that ~32% of the WDR1 RNAi cells (48 h) possessed a detached flagellum (Fig 4D and 4E), suggesting that WDR1 depletion also disrupted flagellum-cell body adhesion in these cells.

The defective segregation of kinetoplasts caused by WDR1 depletion prompted us to investigate the potential defect in basal body duplication/segregation, as kinetoplast segregation is mediated by basal body separation during the cell cycle [32]. Cells were immunostained with anti-TbSAS-6 antibody, which stains the basal body cartwheel [33], to label the mature basal body and the pro-basal body and with anti-PFR2 antibody (L8C4) to stain the flagellum (Fig 4E). The numbers of basal body and flagellum in cells of different cell cycle stages were quantified (Fig 4F). Upon WDR1 RNAi induction for 48 h, there was no significant change in the numbers of basal body and flagellum in 1N1K, 1N2K and 2N2K cells (Fig 4F), but the two pairs of mature basal body/pro-basal body in ~50% of the 2N2K cells were not well segregated as in the control 2N2K cells (Fig 4E). The average distance between the two pairs of mature basal body/pro-basal body in WDR1-deficient 2N2K cells was calculated to be ~3.8 μm, which is significantly shorter than the inter-basal body distance (~5.6 μm) of the control 2N2K cells (Fig 4G). These results suggest defective basal body segregation in WDR1-depleted 2N2K cells. These 2N2K cells also contained a detached flagellum (Fig 4E, black arrow). In the 2N1K cells that accumulated after WDR1 RNAi, although ~30% of them contained two basal bodies (one mature basal body and one pro-basal body) and one flagellum, the rest of them (~70%) contained four basal bodies (two pairs of mature basal body/pro-basal body) and two flagella, but the two pairs of mature basal body/pro-basal body were not well segregated and one of the two flagella was detached (Fig 4E and 4F). The average distance between the two pairs of mature basal body/pro-basal body in the WDR1-deficient 2N1K cells was calculated to be ~2.1 μm, which is less than half of the inter-basal body distance of the control 2N2K cells (Fig 4G). This result suggests that basal body segregation and flagellum attachment in the 2N1K cells was defective.

### Depletion of WDR1 impairs bilobe duplication and disrupts FAZ assembly

**Fig 5:**
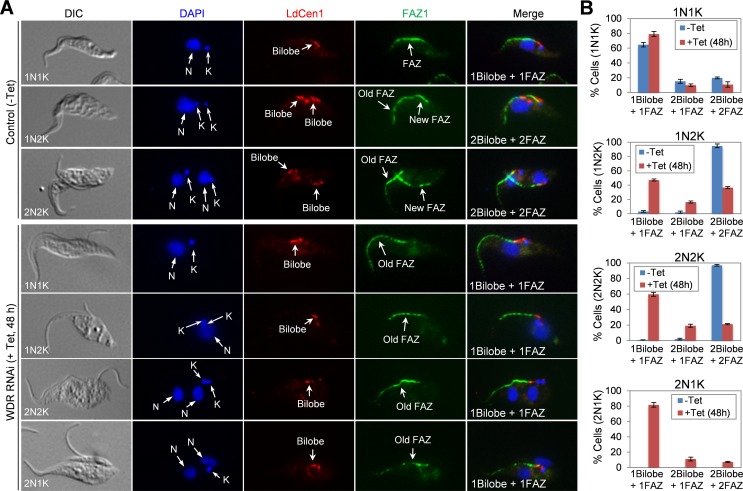
RNAi of WDR1 disrupted bilobe duplication and new FAZ filament assembly. (**A**). Non-induced control cells (-Tet) and WDR1 RNAi-induced cells (+Tet, 48 h) were co-immunostained with L3B2, which labels the FAZ1 protein in the FAZ filament, and LdCen1, which detects TbCentrin4 in the bilobe, and counterstained with DAPI for nuclear (N) and kinetoplast (K) DNA. Scare bar: 5 μm. (**B**). Quantification of cells with different numbers of bilobe and FAZ filament in 1N1K, 1N2K, 2N2K and 2N1K cells. 300 cells were counted for each cell type at each time point, and error bars represent S.D.

We next investigated the effect of WDR1 depletion on the duplication/segregation of the bilobe structure and the assembly of the new FAZ filament. Non-induced control and WDR1 RNAi cells (48 h) were co-immunostained with anti-LdCen1 antibody and anti-FAZ1 antibody (L3B2), which detects FAZ1 in the FAZ filament [34] (Fig 5A). The numbers of bilobe and FAZ filament in cells of different cell cycle stages before and after WDR1 RNAi induction were quantified (Fig 5B). After WDR1 RNAi induction for 48 h, there was a slight increase in the number of 1N1K cells with one bilobe and one FAZ, whereas a significant increase of the 1N2K and 2N2K cells with one bilobe and one FAZ was observed (Fig 5A and 5B). Among the 2N1K cells that emerged upon WDR1 RNAi, the majority (~80%) of them contained one bilobe and one FAZ (Fig 5A and 5B). These results suggest that WDR1 depletion inhibited bilobe duplication and compromised the assembly of the new FAZ filament. Given that FAZ assembly is required for basal body positioning [35] and flagellum-cell body attachment [36,37,38], these results indicate that the defects in basal body segregation and flagellum attachment caused by WDR1 RNAi (Fig 4E and 4F) were very likely attributed to the defect in FAZ assembly.

### Depletion of WDR1 causes TbPLK accumulation in the bilobe and the basal body and continuous phosphorylation of TbCentrin2 at late cell cycle stages

**Fig 6:**
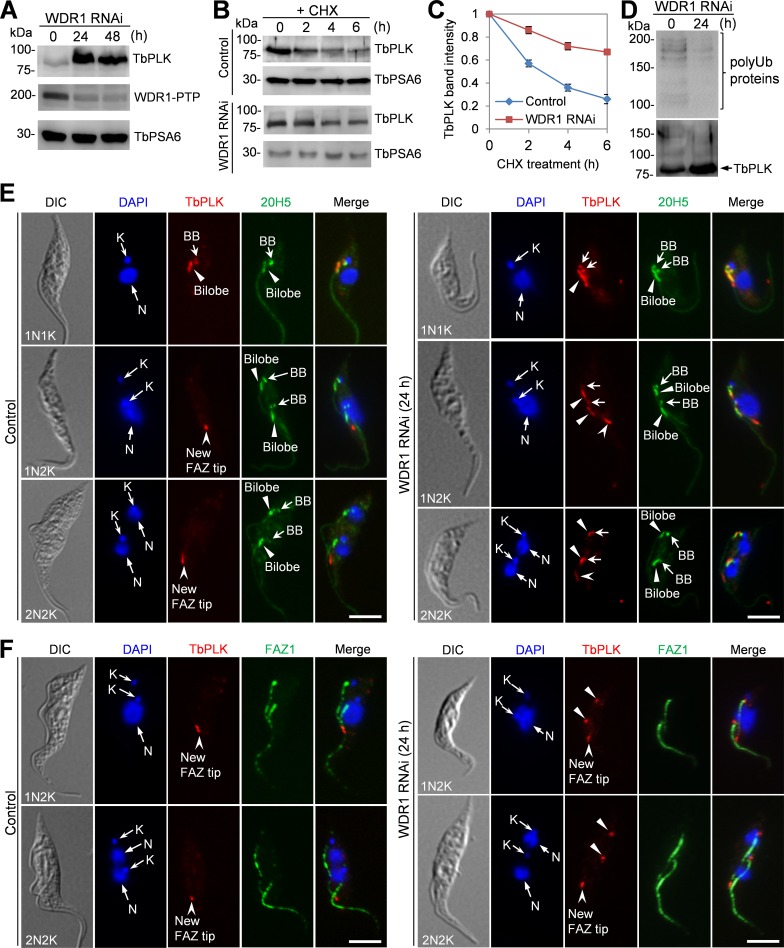
Depletion of WDR1 caused excessive accumulation of TbPLK in the basal body and the bilobe. (**A**). Western blotting to detect the level of TbPLK in control and WDR1 RNAi cells that were induced with tetracycline for 24 and 48 hours. TbPLK was detected by anti-TbPLK antibody, and WDR1 was endogenously tagged with a PTP epitope and detected with anti-Protein A antibody. TbPSA6 served as the loading control. Three repeats were performed, and all showed similar results. (**B**). Degradation of TbPLK in control and WDR1 RNAi cells. The non-induced control cells and WDR1 RNAi cells (24 h) were treated with cycloheximide for up to 6 h, and time course samples were collected at various times after cycloheximide treatment for Western blotting with anti-TbPLK antibody. TbPSA6 served as the loading control. (**C**). Quantification of TbPLK band intensity shown in panel B. TbPLK band intensity was determined with ImageJ, and normalized with the band intensity of TbPSA6. Error bars indicate S.D. calculated from three independent experiments. (**D**). Depletion of WDR1 reduced poly-ubiquitinated proteins of TbPLK immunoprecipitates. TbPLK was immunoprecipitated with anti-TbPLK pAb from non-induced control and WDR1 RNAi cells (24 h), and immunoprecipitated proteins were separated by SDS-PAGE and immunoblotted with anti-ubiquitin mAb and anti-TbPLK pAb to detect poly-ubiquitinated proteins (polyUB proteins) and TbPLK, respectively. Three repeats were performed, which showed similar results. (**E, F**). Effect of WDR1 depletion on TbPLK localization. WDR1 RNAi was induced for 24 h, and cells were co-immunostained with anti-TbPLK pAb and 20H5, which detects centrins in the basal body and the bilobe (panel **E**), and with anti-TbPLK pAb and anti-FAZ1 mAb, which labels the FAZ filament (Panel **F**). The arrows, solid arrowheads and open arrowheads in the TbPLK channel of panel **E** indicate the localization of TbPLK in the basal body, the bilobe and the new FAZ tip, respectively. The solid arrowheads in the TbPLK channel of panel **F** indicate the localization of TbPLK in the bilobe and the basal body region. N, nuclear DNA; K, kinetoplast DNA. Scale bars: 5 μm.

We investigated the effect of WDR1 depletion on TbPLK protein abundance. Western blotting with anti-TbPLK antibody showed that TbPLK protein level was significantly increased after WDR1 was depleted (Fig 6A). Pulse-chase experiments showed that the rate of TbPLK degradation was significantly reduced in WDR1-depleted cells (Fig 6B and 6C). Further, Western blotting of immunoprecipitated TbPLK with anti-ubiquitin antibody showed that poly-ubiquitinated proteins could be detected in the immunoprecipitate of TbPLK and the level of these poly-ubiquitinated proteins in WDR1 RNAi cells was reduced to ~10% of the control level (Fig 6D). However, it should be noted that the identify of these poly-ubiquitinated proteins is unknown because they could include poly-ubiquitinated TbPLK and TbPLK co-precipitated proteins. Together, these results suggest that the increase in TbPLK protein level in WDR1 RNAi cells (Fig 6A) was attributed to the reduction in TbPLK degradation.

TbPLK localizes to the basal body and the bilobe structure during G1 phase of the cell cycle and to the distal tip of the new FAZ thereafter before it disappears from the new FAZ tip at late anaphase [39]. Immunofluorescence microscopy with anti-TbPLK antibody confirmed the dynamic localizations of TbPLK in non-induced control cells (Fig 6E). To investigate the effect of WDR1 RNAi on TbPLK localization, we focused on the cells at the early stage (24 h) of WDR1 RNAi, during which cell cycle progression has not been impaired and cells are still of normal morphology (Figs 4C and 6E). Unlike in the non-induced control 1N2K and 2N2K cells in which TbPLK was only detected at the new FAZ tip (Fig 6E), in all of the WDR1-deficient 1N2K and 2N2K cells TbPLK was detected in the basal body (Fig 6E, arrows) and the bilobe structure (Fig 6E, solid arrowheads), in addition to the new FAZ tip (Fig 6E and 6F, open arrowheads), suggesting the accumulation of TbPLK protein in the basal body and the bilobe in WDR1 RNAi cells. Given the defective TbPLK degradation in WDR1 RNAi cells (Fig 6B and 6C), the accumulation of TbPLK in the basal body and the bilobe suggests that in wild-type cells TbPLK is degraded in the basal body and the bilobe when cells proceed to the S phase of the cell cycle and this degradation requires WDR1.

**Fig 7:**
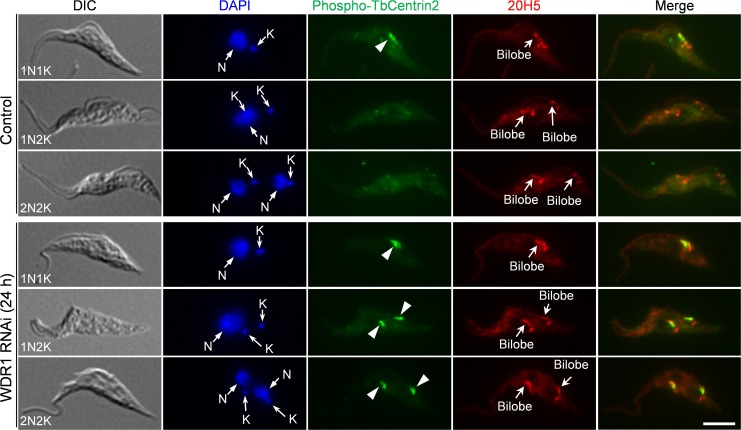
Effect of WDR1 depletion on TbPLK-mediated phosphorylation of TbCentrin2 in the bilobe. Control and WDR1 RNAi cells (24 h) were co-immunostained with PS54 antibody, which detects the phospho-Ser54 of TbCentrin2 (arrowheads), and 20H5, which labels the bilobe. Cells were counterstained with DAPI for nuclear (N) and kinetoplast (K) DNA. Scale bar: 5 μm.

TbPLK is known to phosphorylate TbCentrin2 in the bilobe in 1N1K cells when the bilobe structure is duplicating, and this phosphorylation of TbCentrin2 and subsequent dephosphorylation of TbCentrin2 are required for bilobe duplication and flagellum-cell body adhesion [21]. Given that TbPLK remained in the bilobe in 1N2K and 2N2K cells after WDR1 RNAi (Fig 6E), we speculated that TbCentrin2 should be phosphorylated by TbPLK in these cells. To test this possibility, we immunostained the non-induced control and WDR1 RNAi cells (24 h) with the PS54 antibody, which detects the TbPLK-phosphorylated Ser54 in TbCentrin2 [21]. We confirmed that TbCentrin2 was phosphorylated only in 1N1K cells, but not 1N2K and 2N2K cells in the non-induced control cell population (Fig 7, arrowhead), as reported previously [21]. However, in the 1N2K and 2N2K cells from the WDR1 RNAi cell population, TbCentrin2 was still phosphorylated (Fig 7, arrowheads). These results suggest that bilobe-accumulated TbPLK was able to phosphorylate TbCentrin2 at Ser54 at late cell cycle stages.

### Overexpressed TbPLK accumulates in the basal body and the bilobe, and TbPLK overexpression impairs basal body segregation, FAZ assembly and flagellum attachment

The excessive accumulation of TbPLK protein in WDR1 RNAi cells (Fig 6A) led us to hypothesize that ectopic overexpression of TbPLK would cause defects similar to WDR1 RNAi. Indeed, previous studies showed that overexpression of TbPLK cause a growth defect in the procyclic trypanosomes [19]. Overexpression of TbPLK was confirmed by Western blotting with anti-TbPLK antibody, which showed that TbPLK was overexpressed ~5-fold over the native TbPLK (S4A Fig), similar to that in WDR1 RNAi cells (Fig 6A). Consistent with the previous report [19], this overexpression caused a severe growth defect (S4B Fig), and resulted in an accumulation of 2N1K, XN1K (X>2), XN2K and XNXK cells (S4C Fig). Immunostaining with YL 1/2 antibody and anti-TbSAS-6 antibody showed that ~70% of the 2N1K cells contained two pairs of mature basal body/pro-basal body, which are closely associated around the single kinetoplast (S4D and S4E Fig), suggesting that TbPLK overexpression disrupted basal body segregation. TbPLK overexpression also caused flagellum detachment (S4F and S4G Fig), which was due to the defective assembly of the new FAZ filament (S4F and S4G Fig). Among the 2N1K cells, ~45% of them contained only a full-length old FAZ, and the rest of them contained a full-length old FAZ and a short, new FAZ (snFAZ) (S4F and S4G Fig). Altogether, these results demonstrated that overexpression of TbPLK inhibited basal body segregation and disrupted new FAZ assembly.

Finally, we investigated whether overexpressed TbPLK accumulated in the basal body and the bilobe, as excess TbPLK did in WDR1 RNAi cells (Fig 6E). As a control, localization of the endogenously 3HA-tagged TbPLK was investigated, which showed that endogenous TbPLK-3HA localized to the basal body and the bilobe in 1N1K cells and to the new FAZ tip in 1N2K and 2N2K cells (S4H Fig), identical to the results obtained with anti-TbPLK antibody (Fig 6E). Overexpressed TbPLK-3HA was detected in the basal body and the bilobe in 1N1K cells and in the basal body and the bilobe as well as the new FAZ tip in 1N2K and 2N2K cells (S4H Fig). These results confirmed that overexpressed TbPLK indeed accumulated in the basal body and the bilobe after cell cycle transition to S phase and beyond, providing additional evidence to support that the defects caused by WDR1 RNAi were attributed to the accumulation of TbPLK in the basal body and the bilobe.

## Discussion

**Fig 8:**
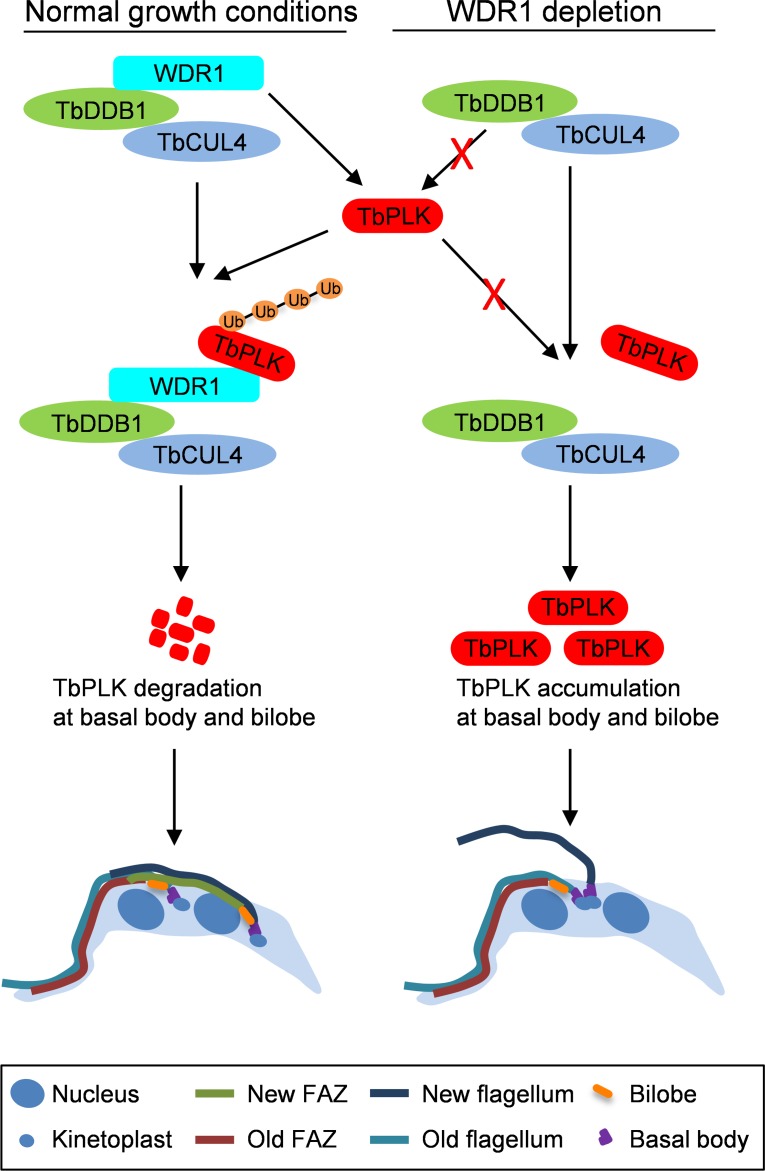
Model of the role of CRL4^WDR1^ in controlling TbPLK protein abundance to promote bilobe duplication, basal body segregation and FAZ assembly. WDR1 in the CRL4 ubiquitin ligase complex recognizes the PEST motif in TbPLK. Binding of TbPLK to WDR1 causes TbPLK ubiquitination by CRL4^WDR1^ and subsequent degradation of TbPLK in the basal body and the bilobe after the G1/S cell cycle transition. This degradation of TbPLK in the basal body and the bilobe promotes bilobe duplication, basal body segregation, FAZ filament assembly, flagellum attachment and faithful cytokinesis. When WDR1 is depleted, TbPLK is not recruited to the TbCUL4-TbDDB1 complex for ubiquitination, leading to TbPLK accumulation in the basal body and the bilobe, where it may continuously phosphorylate the bilobe protein TbCentrin2. This accumulation of TbPLK inhibits bilobe duplication, impairs basal body segregation, disrupts the assembly of the new FAZ filament, which causes flagellum detachment, and blocks cytokinesis.

It has been known for almost a decade that TbPLK protein abundance is tightly controlled, as overexpression of TbPLK is detrimental to trypanosomes [19]. However, how TbPLK protein abundance is regulated was not clear. In this report, we uncovered the mechanism underlying the control of TbPLK protein abundance. We identified a novel Cullin4-containing ubiquitin ligase complex, CRL4^WDR1^, which targets TbPLK in the basal body and the bilobe for degradation by the proteasome after cell cycle transition from G1 to S phase (Fig 8). This may allow dephosphorylation of TbCentrin2 and ensures proper organelle duplication/segregation, FAZ filament assembly, flagellum attachment and cytokinesis. Depletion of WDR1 reduced TbPLK ubiquitination and degradation, leading to excessive accumulation of TbPLK in the basal body and the bilobe and continuous phosphorylation of TbCentrin2, which further caused defective bilobe duplication, FAZ assembly and flagellum attachment (Fig 8). These defects are similar to that caused by expression of the phospho-mimic (constitutively phosphorylated) form of TbCentrin2 in *T*. *brucei* [21]. Our results thus demonstrated that the basal body- and bilobe-localized TbPLK is targeted by CRL4^WDR1^ complex for degradation by the proteasome during the G1 to S phase transition.

Degradation of TbPLK requires both the D-boxes at the N-terminus and the PEST motif located between the kinase domain and the Polo-box domain, but the PEST motif appears to play a dominant role in TbPLK degradation (Fig 1). While the D-box is a characteristic degradation signal for the APC/C ubiquitin ligase [40], the PEST motif in many proteins is recognized by a CRL-type ubiquitin ligase [10,11,41,42,43,44]. Our results demonstrated that CRL4^WDR1^ recognizes the PEST motif in TbPLK, as the PEST deletion mutant (TbPLK-ΔPEST) was not able to bind to WDR1 (Fig 2C) and was stabilized (Fig 1B and 1C). Moreover, we found that the rate of TbPLK degradation in the APC/C subunit Cdc27 RNAi cells was similar to that of TbPLK-DB^mut^ (Fig 1B–1E), suggesting the involvement of APC/C in TbPLK degradation through recognizing the D-boxes. The finding that TbPLK degradation requires both APC/C and a CRL ubiquitin ligase distinguishes TbPLK from its counterparts in humans and fungi, as degradation of human PLK1 and yeast Cdc5 is mediated by APC/C^Cdh1^ [6,7] and degradation of human PLK4 is mediated by SCF^β-TrCP/Slimb^ [8,9]. It is unclear why degradation of TbPLK requires both APC/C and a CRL-type ubiquitin ligase. The trypanosome APC/C has an unusual subunit composition, containing two trypanosome-specific subunit proteins, but it appears to lack the Cdc20/Cdh1 subunit [45]. It remains unclear how TbPLK is recognized by APC/C in the absence of the Cdc20/Cdh1 subunit. Given that WDR1 localizes to the basal body and the bilobe region during G1 and S phases (Fig 2E and 2F) and depletion of WDR1 caused TbPLK accumulation in the basal body and the bilobe (Fig 6E), it suggests that CRL4^WDR1^ may be primarily responsible for TbPLK degradation in the basal body and the bilobe.

CRL-type ubiquitin ligases are the largest family of ubiquitin ligases responsible for degradation of diverse cellular proteins in eukaryotes [31]. Little is known about the CRL complexes in *T*. *brucei*, but bioinformatics analyses identified 6 Cullin homologs, 24 F-box proteins, and 174 WD40-repeat proteins (http://supfam.cs.bris.ac.uk/SUPERFAMILY/cgi-bin/gen_list.cgi?genome=Tb). These F-box proteins and WD40-repeat proteins are potential substrate receptors in the CRL ubiquitin ligases, although their identity remains to be determined experimentally. The CRL4^WDR1^ complex identified in this study is the first CRL-type ubiquitin ligase complex in *T*. *brucei*, and WDR1 appears to act as a substrate receptor, which, through its N-terminal WD40 motif, interacts with TbDDB1 and, through its C-terminal domain, recognizes the PEST sequence of TbPLK (Figs 2C and 3E).

The signal for targeting TbPLK for ubiquitination by CRL4^WDR1^ and subsequent degradation remains unknown. Human PLK4 is trans-autophosphorylated at multiple Ser/Thr residues within the PEST motif, thereby enabling β-TrCP/Slimb binding to the phosphorylated PEST motif and ubiquitination of PLK4 by SCF^β-TrCP/Slimb^ [13,14]. The PEST motif in TbPLK does not contain the characteristic β-TrCP/Slimb-recognition motif (DSGxxT, x denotes any residue) found in the PEST motif of animal PLK4/SAK, but it does contain multiple phosphorylated serine and threonine residues, including Thr451, Ser462, Thr465, Thr466, Thr468 and Thr469 (Fig 1A) [24]. However, mutation of these serine and threonine residues to alanine did not stabilize TbPLK (Fig 1B and 1C), suggesting that phosphorylation of the PEST motif in TbPLK does not serve as the signal for targeting TbPLK for ubiquitination and degradation. Moreover, inhibition of TbPLK activity by the small molecular inhibitor GW843682X did not stabilize TbPLK (S1D Fig), suggesting that autophosphorylation of TbPLK does not contribute to TbPLK degradation. Together, these results suggest that the regulation of TbPLK stability likely involves a mechanism distinct from that for the animal PLK4.

The defects caused by WDR1 RNAi and TbPLK overexpression raised an interesting question of how the accumulation of TbPLK in the bilobe and the basal body inhibited bilobe duplication, basal body segregation and FAZ assembly. TbPLK is known to phosphorylate TbCentrin2 in the bilobe [21] and SPBB1 in the basal body [20]. The role of TbPLK phosphorylation of SPBB1 remains unknown, but TbPLK phosphorylation of TbCentrin2 appears to be essential for TbCentrin2 function. Importantly, expression of a phospho-mimic TbCentrin2 mutant in *T*. *brucei* caused stronger defects in bilobe duplication and FAZ assembly than the phospho-deficient TbCentrin2 mutant, indicating that dephosphorylation of TbCentrin2 is required for bilobe duplication and FAZ assembly [21]. The fact that WDR1 depletion caused phosphorylation of TbCentrin2 in 1N2K and 2N2K (Fig 7) suggests that the defects in bilobe duplication and FAZ assembly in WDR1 RNAi cells are attributed, at least partly, to the lack of dephosphorylation of TbCentrin2. Assembly of the new FAZ filament occurs at the proximal region of the bilobe [38,46] and appears to depend on bilobe duplication [47]. Moreover, segregation of the duplicated basal bodies has been demonstrated to be dependent on the assembly and elongation of the new FAZ filament [35]. Given that bilobe duplication is required for FAZ assembly and FAZ assembly/elongation promotes basal body segregation, it is very likely that the inhibition of bilobe duplication in WDR1 RNAi cells was the primary defect, which led to defective FAZ assembly. Further, failure to assemble the new FAZ filament inhibited basal body segregation and caused flagellum detachment.

In summary, our results suggest that the CRL4^WDR1^ complex targets bilobe- and basal body-localized TbPLK for degradation after cells enter the S phase of the cell cycle, thereby preventing continuous phosphorylation of the bilobe protein TbCentrin2 to ensure bilobe duplication, basal body segregation, FAZ assembly and flagellum-cell body adhesion. This finding uncovered the mechanism underlying the tight control of TbPLK protein abundance.

## Material and Methods

### Trypanosome cell culture and RNAi

The procyclic form of *T*. *brucei* strain 29–13 [48] was cultured at 27°C in SDM-79 medium supplemented with 10% fetal bovine serum (Atlanta Biologicals, Inc, GA, USA), 15 μg/ml G418 and 50 μg/ml hygromycin B. The procyclic strain Lister427 was maintained in SDM-79 medium containing 10% fetal bovine serum.

To knock down WDR1 by RNAi, two DNA fragments (nucleotides 3 to 519 and nucleotides 1251 to 1740) of *WDR1* gene were PCR amplified from the genomic DNA and cloned into the pZJM vector [49]. The resulting plasmids were linearized by *Not* I restriction digestion and electroporated into the 29–13 cell line. Transfectants were selected under 2.5 μg/ml phleomycin and cloned by limiting dilution in a 96-well plate containing the SDM-79 medium supplemented with 20% fetal bovine serum and appropriate antibiotics. Two clonal cell lines from each WDR1 RNAi cell line were selected and analyzed. The Cdc27 RNAi cell line was described previously [25]. RNAi was induced by incubating the clonal cell lines with 1.0 μg/ml tetracycline.

### Overexpression of TbPLK and its various mutants

To overexpress TbPLK in *T*. *brucei*, TbPLK was cloned into pLew100-3HA [50] and transfected into the 29–13 cell line. To make PEST-deletion mutant, the sequences flanking the PEST motif (a.a. 448–471) were fused in frame and cloned into the pLew100-3HA vector. To mutate Arg74, Arg285 and Arg375 to alanine in the D-boxes of TbPLK, site-directed mutagenesis was performed with pLew100-TbPLK-3HA and pLew100-TbPLK-ΔPEST-3HA as the templates to make the D-box mutant (TbPLK-DB^mut^) and D-box and PEST double mutant (TbPLK-DB^mut^-ΔPEST). Site-directed mutagenesis was also carried out to mutate the serine and threonine residues in the PEST motif to alanine, which generated the phosphodeficient PEST mutant of TbPLK (TbPLK-ST/AA). Mutation of the D-boxes and the serine and threonine residues, and deletion of the PEST sequence were verified by sequencing. The plasmids were linearized with *Not* I and electroporated into the 29–13 cell line. The transfectants were selected under 2.5 μg/ml phleomycin and cloned by limiting dilution in a 96-well plate. Overexpression of TbPLK and its various mutants was induced by 1.0 μg/ml tetracycline.

### Directional yeast two-hybrid assay

DNA fragments encoding the N- and C-terminal truncations of WDR1 were cloned into the pGAD-T7 vector, and the resulting constructs were transformed into yeast strain AH109. pGBK-TbPLK-K70R and pGBK-PBD_TbPLK_ [20,50] were transformed into yeast strain Y187. Yeast mating was performed as reported previously [20,50]. The diploid yeast strains were spotted onto SD plates without Trp and Leu (double drop-out) and SD plates without Trp, Leu and His (triple drop-out). Growth of yeast on the triple drop-out plate indicates interaction between the bait and the pray proteins.

### In vitro GST pull-down assays

DNA fragments encoding the N- and C-terminal truncations of WDR1 were cloned into the pGEX-4T-3 vector, and the resulting constructs (pGEX-WDR1^1-300^, pGEX-WDR1-B and pGEX-WDR1-C) were transformed into *E*. *coli* BL21 strain for expressing GST-fused WDR1^1-300^, WDR1-B and WDR1-C proteins. Affinity purified GST-WDR1-B and GST-WDR1-C were bound to glutathione sepharose beads and incubated with trypanosome cell lysate containing overexpressed TbPLK-3HA, TbPLK-K70R-3HA or TbPLK-ΔPEST-3HA. Affinity purified GST-WDR1^1-300^ was bound to glutathione sepharose beads and incubated with *T*. *brucei* cell lysate containing TbDDB1-3HA. The beads were then washed six times with wash buffer (25 mM Tris-HCl, pH 7.6, 150 mM NaCl, 1 mM DTT, 1% Nonidet P-40 and protease inhibitor cocktail), and bound proteins were eluted with 10% SDS, separated by SDS-PAGE and immunoblotted with anti-HA mAb. Purified GST was used as the negative control. Purified GST-fusion proteins and GST were stained with coomassie brilliant blue.

### Epitope tagging of endogenous proteins

WDR1 was endogenously tagged at the C-terminus with a triple HA epitope or a PTP epitope using the PCR-based one-step tagging approach [51], in both Lister427 cell line and WDR1 RNAi cell line. Transfectants were selected under 1.0 μg/ml puromycin and cloned by limiting dilution in a 96-well plate.

To tag the five Cullin proteins and TbDDB1 with a PTP epitope in their respective endogenous locus, DNA fragments corresponding to the C-terminal region of TbCUL1 (545-bp), TbCUL2 (750-bp), TbCUL3 (609-bp), TbCUL4 (780-bp), TbCUL6 (1025-bp) and TbDDB1 (657-bp) were each cloned into the pC-PTP-NEO vector. The resulting plasmids were linearized with *Xcm* I (for TbCUL1, TbCUL2 and TbCUL3), *Mfe* I (for TbCUL4 and TbCUL6), and *Xho* I (for TbDDB1), respectively, and then transfected into the cells expressing WDR1-3HA. The transfectants were selected under 40 μg/ml G418 in addition to 1 μg/ml puromycin and cloned by limiting dilution.

To generate the cell line co-expressing WDR1-3HA, TbCUL4-PTP and TbDDB1-3Myc, the one-step PCR-based protein tagging approach [51] was carried out to tag TbDDB1 with a triple Myc epitope in the cell line that co-expresses WDR1-3HA and TbCUL4-PTP (see above). The transfectants were selected under 10 μg/ml Blasticidin in addition to 40 μg/ml G418 and 1 μg/ml puromycin, and cloned by limiting dilution in a 96-well plate.

### Co-immunoprecipitation

Cells (10^7^) expressing 3HA-tagged WDR1 from its endogenous locus were harvested by centrifugation, lysed in 1 ml immunoprecipitation buffer (25 mM Tris-HCl, pH 7.6, 150 mM NaCl, 1 mM DTT, 1% Nonidet P-40 and protease inhibitor cocktail), and incubated with 20 μl anti-HA antibody conjugated to protein G sepharose beads at 4°C for 1 h. The beads were then washed 6 times with the immunoprecipitation buffer, and bound proteins were eluted with 10% SDS, separated by SDS-PAGE, transferred onto a PVDF membrane and immunoblotted with anti-TbPLK pAb and anti-HA mAb to detect TbPLK and WDR1-3HA, respectively. Wild-type 427 cells were included as the negative control.

Cells (10^7^) co-expressing 3HA-tagged WDR1 and each of the five PTP-tagged Cullin proteins or PTP-tagged TbDDB1 were lysed in 1 ml immunoprecipitation buffer (see above), and the cleared lysate was incubated with 20 μl IgG sepharose beads (GE HealthCare) at 4°C for 1 h. The beads were then washed 6 times with the immunoprecipitation buffer, and bound proteins were eluted with 10% SDS, separated by SDS-PAGE, transferred onto a PVDF membrane and immunoblotted with anti-HA mAb and anti-Protein A pAb to detect WDR1-3HA and TbCUL4-PTP and TbDDB1-PTP, respectively. Cells expressing WDR1-3HA alone were included as the negative control to rule out the possibility that the IgG beads may bind to the triple HA epitope.

For co-immunoprecipitation of WDR1-3HA, TbCUL4-PTP, TbDDB1-3Myc and TbPLK, cells co-expressing WDR1-3HA, TbCUL4-PTP and TbDDB1-3Myc were lysed, and the cleared lysate was incubated with 20μl EZview Red anti-HA affinity gel (Sigma-Aldrich). Immunoprecipitated proteins were eluted from the beads, separated by SDS-PAGE and immunoblotted with anti-HA antibody, anti-Myc antibody, anti-TbPLK antibody and anti-Protein A antibody to detect WDR1-3HA, TbDDB1-3Myc, TbPLK and TbCUL4-PTP, respectively.

For immunoprecipitation of poly-ubiquitinated TbPLK, control and WDR1 RNAi cells (24 h) were lysed, and the cleared lysate was incubated with anti-TbPLK pAb and then with protein A sepharose beads. Immunoprecipitated proteins were eluted from the beads, separated by SDS-PAGE and immunoblotted with anti-Ubiquitin mAb (Sigma-Aldrich) and anti-TbPLK antibody to detect poly-ubiquitinated TbPLK and TbPLK, respectively.

### Immunofluorescence microscopy

Cells were washed with PBS, adhered onto coverslips and fixed in cold (-20°C) methanol for 30 min. Fixed cells were rehydrated by washing with PBS, and then incubated in blocking buffer (3% BSA in PBS) for 1 h at room temperature. Cells were incubated with the primary antibody diluted in PBS containing 3% BSA at room temperature for 1 h. The following primary antibodies were used: FITC-conjugated anti-HA mAb (1:400 dilution, Sigma-Aldrich), anti-Protein A pAb (1: 400 dilution, Sigma-Aldrich), anti-TbPLK pAb (1:1,000 dilution) [20], anti-TbSAS-6 pAb (1:400 dilution) [33], L3B2 (anti-FAZ1 mAb, 1:20 dilution) [34], YL 1/2 (1:1,000 dilution) [52], 20H5 (anti-Centrin mAb, 1:400 dilution) [53], PS54 (anti-phospho-Ser54 of TbCentrin2 pAb, 1: 30,000 dilution) [21], L8C4 (anti-PFR2 mAb, 1:50 dilution) [34], and anti-LdCen1 pAb (1:400 dilution) [26]. After three washes with wash buffer (0.1% Triton X-100 in PBS), cells were incubated with FITC-conjugated or Cy3-conjugated secondary antibody at room temperature for another hour. The following secondary antibodies were used: FITC-conjugated anti-mouse IgG (Sigma-Aldrich), FITC-conjugated anti-rat IgG (Sigma-Aldrich), Cy3-conjugated anti-rabbit IgG (Sigma-Aldrich), Cy3-conjugated anti-mouse IgG (Sigma-Aldrich). After three more washes with the wash buffer, the slides were mounted in VectaShield mounting medium (Vector Labs, CA, USA) containing DAPI and examined with an inverted fluorescence microscope (Olympus IX71) equipped with a cooled CCD camera (model Orca-ER, Hamamatsu) and a PlanApo N 60× 1.42-NA DIC objective. Images were acquired using the Slidebook5 software (Intelligent Imaging Innovations).

## Supporting Information

S1 Fig Localization of various TbPLK mutants and degradation of TbPLK D-box and PEST double mutant. (**A**). Cells overexpressing various TbPLK mutants were fixed with cold methanol and then immunostained with FITC-conjugated anti-HA antibody to detect TbPLK mutant proteins tagged with a triple HA epitope. Scale bar: 5 μm. (**B**). Pulse-chase experiment to monitor the degradation of TbPLK-DB^mut^-ΔPEST. Cells overexpressing 3HA-tagged TbPLK-DB^mut^-ΔPEST were treated with cycloheximide, and time-course samples were collected for Western blotting with anti-HA antibody. In a separate cell sample, MG-132 was added together with cycloheximide and incubated for 12 h (12h+MG-132). TbPSA6 served as the loading control. (**C**). Quantification of TbPLK-DB^mut^-ΔPEST band intensity from panel B. TbPLK-DB^mut^-ΔPEST band intensity was measured with ImageJ, and normalized with that of TbPSA6. Error bars represent S.D. calculated from three independent experiments. (**D**). Pulse-chase experiment to monitor the degradation of TbPLK in the presence of TbPLK inhibitor GW843682X. Cells were treated with GW843682X for 16 h, and then additionally treated with cycloheximide for 12 h. Time-course samples were collected for Western blotting with anti-TbPLK antibody. In a separate cell sample, MG-132 was added together with cycloheximide and GW843682X and incubated for 12 h (12h+MG-132). TbPSA6 served as the loading control.
(PDF)

S2 Fig Sequence alignment of TbCUL4 with its counterparts from fission yeast and humans. Tb, *Trypanosoma brucei*; *Sp*, *Schizosaccharomyces pombe*; Hs, *Homo sapiens*.
(PDF)

S3 Fig Sequence alignment of TbDDB1 with its counterparts from fission yeast and humans. Tb, *Trypanosoma brucei*; *Sp*, *Schizosaccharomyces pombe*; Hs, *Homo sapiens*.
(PDF)

S4 Fig Overexpressed TbPLK accumulated in the basal body and the bilobe, and TbPLK overexpression impaired basal body segregation, FAZ assembly and flagellum attachment. (**A**). Overexpression of TbPLK-3HA. Shown are the Western blots of control and tetracycline-induced cells with anti-TbPLK pAb, anti-HA antibody, and anti-TbPSA6 pAb, which served as the loading control. (**B**). Overexpression of TbPLK caused a severe growth defect. (**C**). Effect of TbPLK overexpression on cell cycle progression. (**D**). Overexpression of TbPLK inhibited basal body segregation. Cells were immunostained with YL 1/2 mAb to label the mature basal body (arrows) and anti-TbSAS-6 pAb to stain the mature basal body and the pro-basal body. N, nuclear DNA; K, kinetoplast DNA. Scale bar: 5 μm. (**E**). Quantification of mature basal body (mBB) and total basal body (BB, mBB and pBB) in 2N1K cells. X>2. 200 cells were counted, and error bars indicate S.D. (**F**). TbPLK overexpression impaired FAZ assembly, leading to flagellum detachment. Cells were immunostained with anti-FAZ1 antibody (L3B2). snFAZ, short, new FAZ; DF: detached flagellum; AF: attached flagellum. Scale bar: 5 μm. (**G**). Quantification of FAZ filaments, detached flagellum (DF) and attached flagellum (AF) in 2N1K cells. 200 cells were counted, and error bars indicate S.D. (**H**). Localization of endogenously tagged TbPLK-3HA and overexpressed TbPLK-3HA. Cells were co-immunostained with FITC-conjugated anti-HA antibody and anti-LdCen1 antibody, which labels the basal body (BB) and the bilobe structure. Scale bar: 5 μm.
(PDF)
