# Supplementary figures and images for: CRL4WDR1 Controls Polo-like Kinase Protein Abundance to Promote Bilobe Duplication, Basal Body Segregation and Flagellum Attachment in Trypanosoma brucei
Source: PLoS Pathog. 2017 Jan 4;13(1):e1006146. doi: 10.1371/journal.ppat.1006146 (PMC5241021; doi:10.1371/journal.ppat.1006146)

S1 Figure

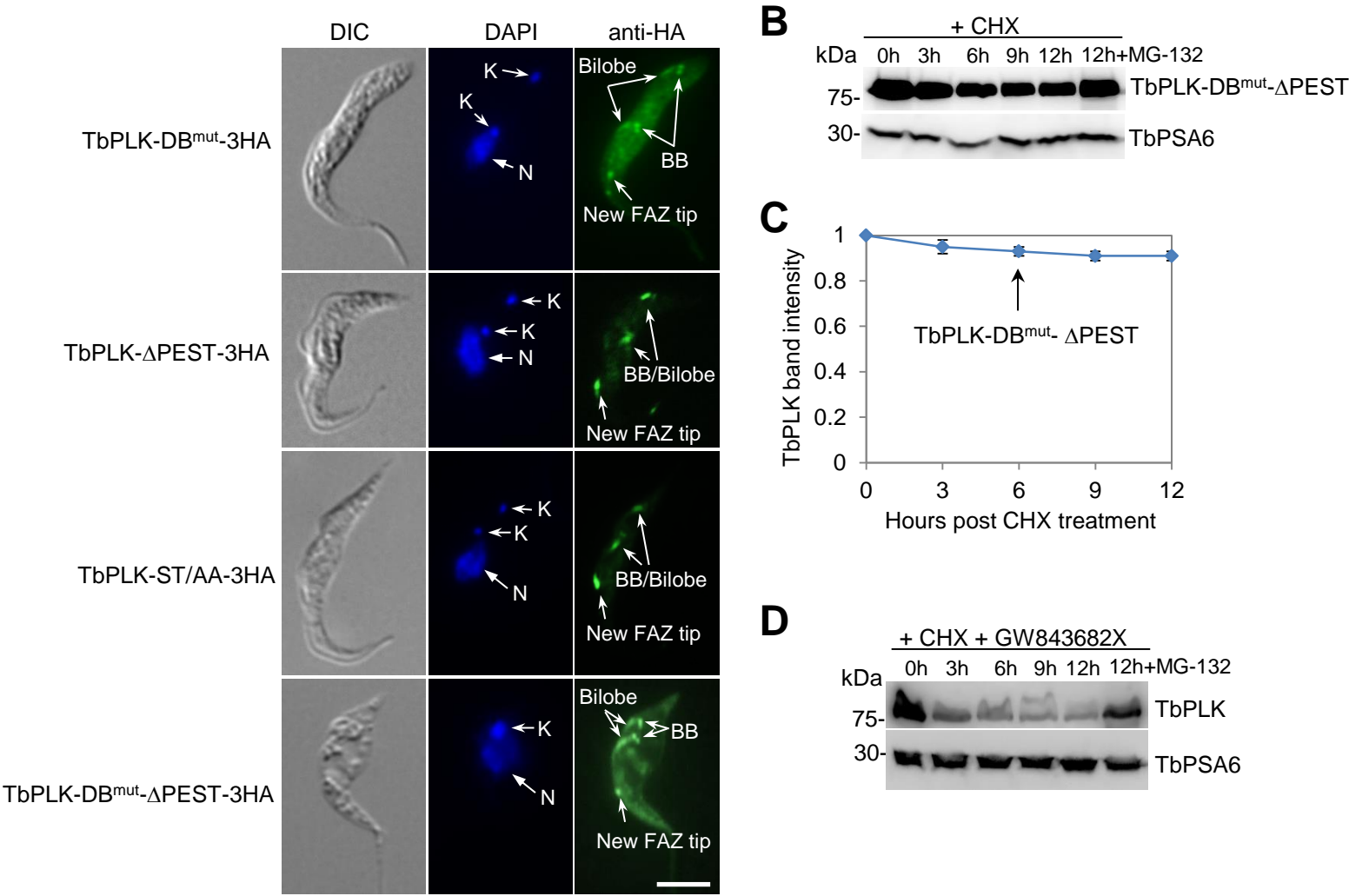

Supplement: S1 Fig — (A). Cells overexpressing various TbPLK mutants were fixed with cold methanol and then immunostained with FITC-conjugated anti-HA antibody to detect TbPLK mutant proteins tagged with a triple HA epitope. Scale bar: 5 μm. (B). Pulse-chase experiment to monitor the degradation of TbPLK-DBmut-ΔPEST. Cells overexpressing 3HA-tagged TbPLK-DBmut-ΔPEST were treated with cycloheximide, and time-course samples were collected for Western blotting with anti-HA antibody. In a separate cell sample, MG-132 was added together with cycloheximide and incubated for 12 h (12h+MG-132). TbPSA6 served as the loading control. (C). Quantification of TbPLK-DBmut-ΔPEST band intensity from panel B. TbPLK-DBmut-ΔPEST band intensity was measured with ImageJ, and normalized with that of TbPSA6. Error bars represent S.D. calculated from three independent experiments. (D). Pulse-chase experiment to monitor the degradation of TbPLK in the presence of TbPLK inhibitor GW843682X. Cells were treated with GW843682X for 16 h, and then additionally treated with cycloheximide for 12 h. Time-course samples were collected for Western blotting with anti-TbPLK antibody. In a separate cell sample, MG-132 was added together with cycloheximide and GW843682X and incubated for 12 h (12h+MG-132). TbPSA6 served as the loading control. (PDF) [file ppat.1006146.s001.pdf]

S2 Figure

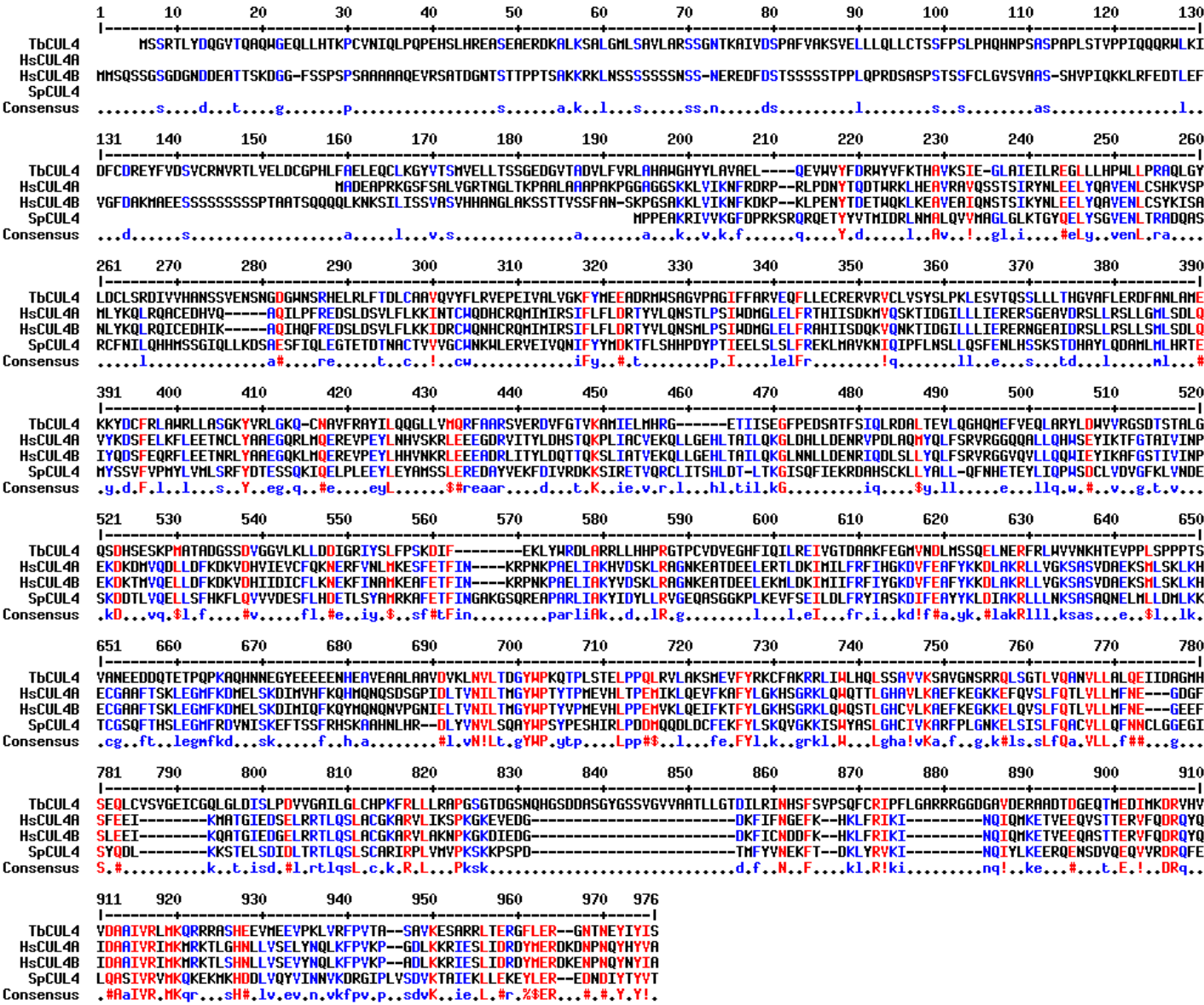

Supplement: S2 Fig — Tb, Trypanosoma brucei; Sp, Schizosaccharomyces pombe; Hs, Homo sapiens. (PDF) [file ppat.1006146.s002.pdf]

S3 Figure

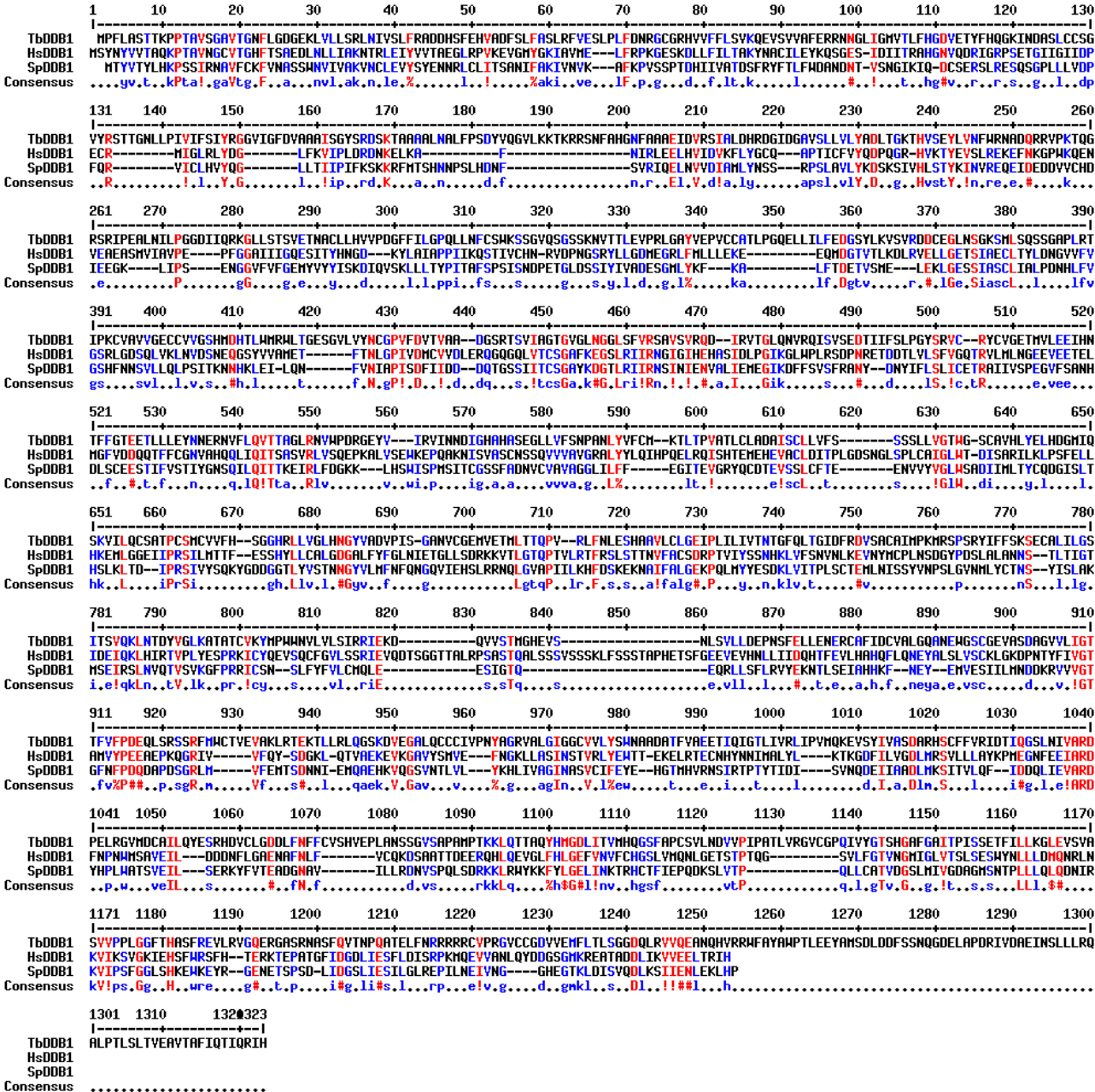

Supplement: S3 Fig — Tb, Trypanosoma brucei; Sp, Schizosaccharomyces pombe; Hs, Homo sapiens. (PDF) [file ppat.1006146.s003.pdf]

**A**

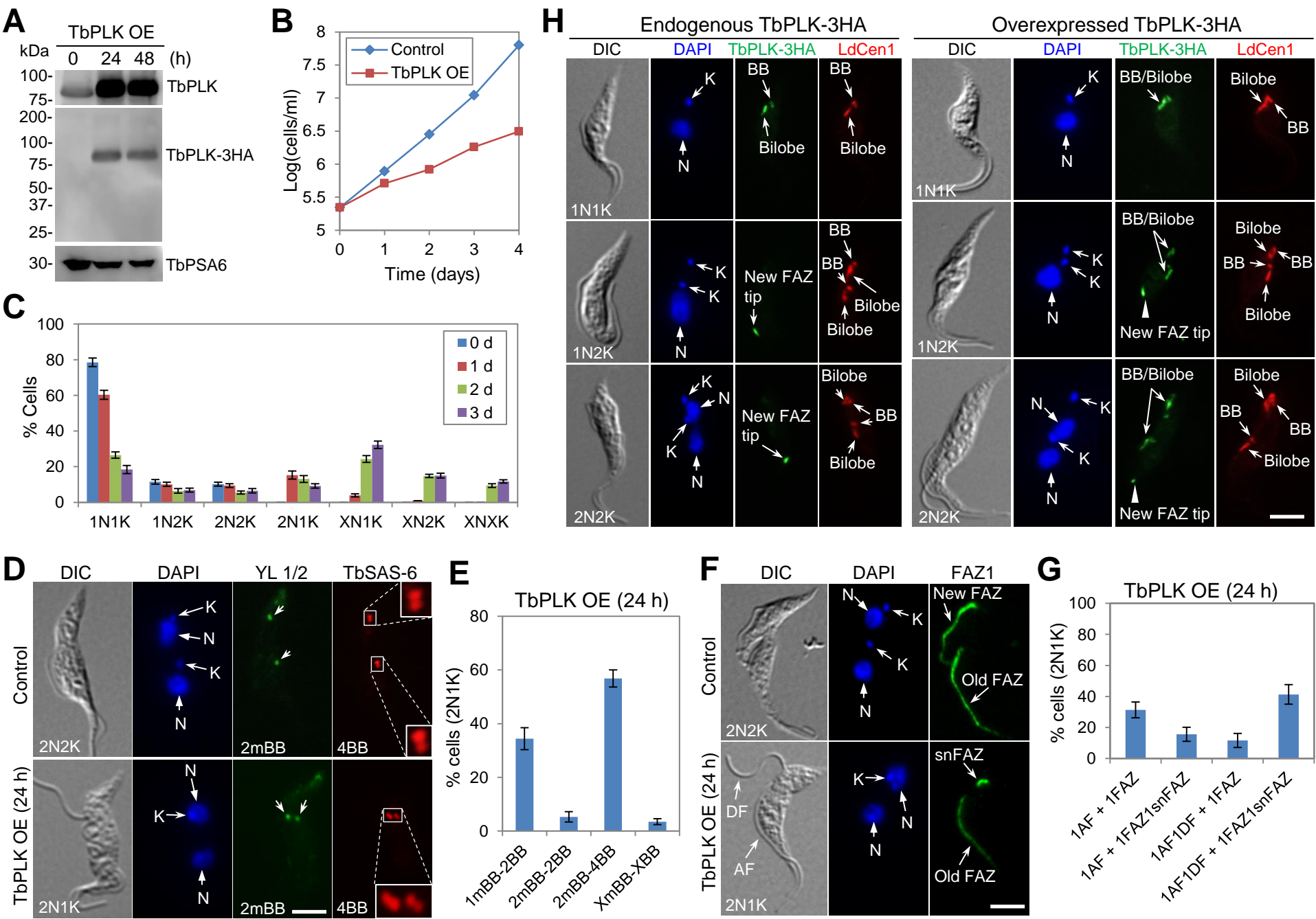

Supplement: S4 Fig — (A). Overexpression of TbPLK-3HA. Shown are the Western blots of control and tetracycline-induced cells with anti-TbPLK pAb, anti-HA antibody, and anti-TbPSA6 pAb, which served as the loading control. (B). Overexpression of TbPLK caused a severe growth defect. (C). Effect of TbPLK overexpression on cell cycle progression. (D). Overexpression of TbPLK inhibited basal body segregation. Cells were immunostained with YL 1/2 mAb to label the mature basal body (arrows) and anti-TbSAS-6 pAb to stain the mature basal body and the pro-basal body. N, nuclear DNA; K, kinetoplast DNA. Scale bar: 5 μm. (E). Quantification of mature basal body (mBB) and total basal body (BB, mBB and pBB) in 2N1K cells. X>2. 200 cells were counted, and error bars indicate S.D. (F). TbPLK overexpression impaired FAZ assembly, leading to flagellum detachment. Cells were immunostained with anti-FAZ1 antibody (L3B2). snFAZ, short, new FAZ; DF: detached flagellum; AF: attached flagellum. Scale bar: 5 μm. (G). Quantification of FAZ filaments, detached flagellum (DF) and attached flagellum (AF) in 2N1K cells. 200 cells were counted, and error bars indicate S.D. (H). Localization of endogenously tagged TbPLK-3HA and overexpressed TbPLK-3HA. Cells were co-immunostained with FITC-conjugated anti-HA antibody and anti-LdCen1 antibody, which labels the basal body (BB) and the bilobe structure. Scale bar: 5 μm. (PDF) [file ppat.1006146.s004.pdf]
